# EVALUATION OF THE IMPACT OF COMMUNITY CARE IN KOREA: NAMYANGJU CITY’S FOUR-YEAR EXPERIENCE

**DOI:** 10.1093/geroni/igad104.2896

**Published:** 2023-12-21

**Authors:** Mi Sun Choi, Sangmi Cho, Yoonsei Park, Misuk Woo, Sumin Park, Youngmin Cho

**Affiliations:** Silla University, Busan, Republic of Korea; Ewha Womans University, Seoul, Republic of Korea; Ewha Womans University, Seoul, Republic of Korea; Ewha Womans University, Seoul, Republic of Korea; Ewha Womans University, Seoul, Republic of Korea; Ewha Womans University, Seoul, Republic of Korea

## Abstract

In 2019, the Korean government launched a community care project in response to the rising need for care brought on by the nation’s aging population and state-funded medical expenses. Namyangju, Korea, has implemented community care for the care of older people. However, little is known about how to ensure the sustainability of community care by investigating the impact of community care services. To enhance our knowledge in this domain, we conducted focus group interviews (FGI) with 24 participants, including 18 service providers and six older adult service users, from August to November 2022. In the FGI, service providers and users identified vehicle and meal support services as core needs. They described the beneficial outcomes of these services, such as increased psychological well-being and the desire for independence and decreased depression. On the other hand, users expressed difficulty in accessing information about various services and mentioned restrictions on service usage (period, number of times), while the providers emphasized the difficulty of providing comprehensive services when health and welfare services were separated amid budget constraints. Both groups proposed solutions to these problems through community care services. Individual citizens’ perceptions of universal welfare should be changed, while institutions must actively promote this program and recruit professionals. A partnership between private and public institutions must be established at the regional level, and services must be differentiated to reflect the region’s distinctive features. Finally, national legislation must be enacted to promote care coordinators and ensure that appropriate budgets are allocated for successfully operating the program.

